# Sub-Surface Defect Depth Approximation in Cold Infrared Thermography

**DOI:** 10.3390/s22187098

**Published:** 2022-09-19

**Authors:** Siavash Doshvarpassand, Xiangyu Wang

**Affiliations:** 1 Australasian Joint Research Centre for Building Information Modelling, Curtin University, Bentley, WA 6102, Australia; 2 WA School of Mines, Curtin University, Bentley, WA 6102, Australia; 3 School of Civil Engineering and Architecture, East China Jiaotong University, Nanchang 330013, China

**Keywords:** cold infrared thermography, non-destructive testing, metal loss defect characterisation, defect depth prediction, structural health monitoring, vision-based sensors

## Abstract

Detection and characterisation of hidden corrosion are considered challenging yet crucial activities in many sensitive industrial plants where preventing the loss of containment or structural reliability are paramount. In the last two decades, infrared (IR) thermography has proved to be a reliable means for inspection of corrosion or other sub-surface anomalies in low to mid thickness metallic mediums. The foundation of using IR thermography for defect detection and characterisation is based on active thermography. In this method of inspection, an external excitation source is deployed for the purpose of stimulating thermal evolutions inside objects. The presence of sub-surface defects disrupts the evolution of electromagnetic pulse inside an object. The reflection of altered pulse at the surface can be recorded through thermal camera in the form of temperature anomalies. Through authors’ previous works, cold thermography has shown that it can be a viable defect detection alternative to the most commonly used means of active thermography, known as heating. In the current work, the characterisation of defect dimensions, i.e., depth and diameter, has been explored. A simple analytical model for thermal contrast over defect is used in order to approximate the defect depth and diameter. This is achieved by comparing the similarities of the model and the experimental contrast time-series. A method of time-series similarity measurement known as dynamic time wrapping (DTW) is used to score the similarity between a pair of model and experiment time-series. The final outcome of the proposed experimental setup has revealed that there is a good potential to predict the metal loss of up to 50% in mid-thickness substrate even by deploying a less accurate nonradiometric thermal device and no advanced image processing.

## 1. Introduction

Detecting and characterising hidden corrosion are considered to be challenging tasks in many sensitive industrial plants where preventing the loss of containment or structural reliability are paramount. An unattended event, such as a prolonged corrosion, can impose significant threat to projects’ reliability, economy and their subsequent operations [1,2,3,4,5,6,7,8,9]. General corrosion characterisation in the form of measuring the material loss forms a dominant activity in corrosion control and risk-based inspections [10,11,12,13,14,15,16,17,18,19]. In the last two decades, infrared (IR) thermography proved to be a reliable means for inspection of corrosion or other sub-surface anomalies in low to mid thickness metals. Infrared thermography, thermal imaging or, in general, thermography is considered a non-destructive examination process that allows observation of the heat patterns over an object surface [20].

Active thermography is a thermography technique in which an external excitation source is deployed for the purpose of stimulating the thermal evolutions inside objects. This energy source can be either physical (stimulating internal energy by causing internal vibration, e.g., microwave or electromagnetic eddy current), optical (e.g., pulse heating using flash lamps, etc.) or, in general, by heat conduction means (heating or cooling objects). Thermal camera records both the temporal and spatial evolutions of surface temperature from the stimulation moment till stabilising to the ambient temperature. Active thermography constitutes a considerable part of thermographic condition monitoring activities accounting for hidden defect detection, i.e., disbonding/delamination defect detection in composites or corrosion/metal loss/crack defect detection in metallic materials. Management and mitigation of metal loss/corrosion defect across metallic components and equipment require ongoing and in situ inspection and monitoring techniques to assure the continuous record of equipment health status [1,21]. As previously noted, very few reports of using cooling mechanisms instead of heating have been mentioned in the body of literature [1,22,23].

**Figure 1:**
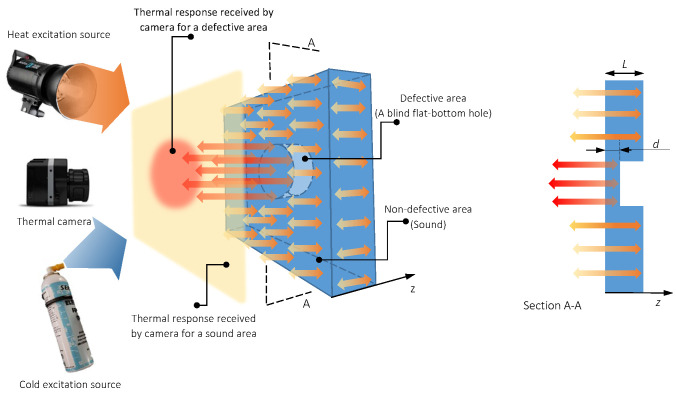
Schematic of thermal diffusion and response through a defective solid, retrieved from [1].

The physical basis of defect detection by means of active thermography is based on the thermal conduction (diffusion) phenomena in solids. Solving the thermal wave equation known as *Fourier* equation for certain boundary conditions leads to estimation of important trends, e.g., temporal temperature evolution. Considering uniform stimulation of a sample surface, thermal propagation into the body of the sample has been classically modelled as a one-dimensional heat-flow process [24,25,26]. 1D heat flow then is governed by simplifying the Fourier equation as follows:

(1) $$\frac{\partial T}{\partial t}=\alpha \frac{\partial^{2}T}{\partial z^{2}}$$

where α=k/ρC is thermal diffusivity $\left(m^{2}/s\right)$. k is thermal conductivity (W/mK); ρ is density $\left(\mathrm{kg}/m^{3}\right)$ and C is specific heat (J/kg K). Sub-surface defects will disrupt the evolution of energy wave propagated inside objects. Consequently, defects will appear in the form of anomalies with different temperature or colour intensities compared to the surrounding sound (non-defective) areas in the infrared detector (see Figure 1).

The solution of Equation (1) for a Dirac delta pulse (an intense unit-area pulse of so short a duration that no measuring equipment is capable of differentiating it from a shorter pulse [27,28]) from a plane source of strength $J_{0}/\rho C$ launched from the surface (z = 0) of a semi-infinite medium (z >> 0) was proposed as follows [24]:

(2) $$T_{semi-inf}\left(z,\;t\right)=T_{0}+\frac{J_{0}}{e\sqrt{\pi t}}exp\left(\frac{-z^{2}}{4\alpha t}\right)$$

considering initial condition of:

(3) $$T\left(z,\;t\right)=T_{0}\;{|}_{t=0}$$

and boundary condition of:

(4) $$\frac{\partial T}{\partial z}=0\;{|}_{z=0}$$

where $T_{semi-inf}$ corresponds to the temperature evolution in the semi-infinite body, $T_{0}$ is initial (ambient) temperature, $J_{0}$ is the thermal energy density $\left(J/m^{2}\right)$ and $e=\sqrt{\rho kC}$ is thermal effusivity $\left({\mathrm{Ws}}^{1/2}/m^{2}k\right)$. From Equation (2), the surface (z=0) temperature of semi-infinite homogenous and opaque medium decays with $1/\sqrt{t}$. Any changes to this characteristic result in variation in temperature evolution at the surface, which can reflect regions containing sub-surface defects.

Through the literature, corrosion defect is generally modelled as a semi-infinite air gap located beneath the surface of a single-layered solid by a distance L and in a plane parallel to the surface. Therefore, the measurement of corrosion is reduced to measurement of the local wall thickness, L [29,30]. The air gap is characterised with much less thermal conductivity than the solid and it will reflect the most of the incident thermal energy, received from a pulse, back to the surface [26]. The thermal reflection coefficient, Γ, of the interface defect is equivalent to [29,31]:

(5) $$\Gamma =\frac{e_{m}-e_{d}}{e_{m}+e_{d}}$$

where $e_{m}$ and $e_{d}$ are, respectively, the thermal effusivity of solid material and defect. In case of corrosion defects modelled as air gap defect, Γ=1 is a valid approximation considering $e_{m}\gg e_{d}$.

A representation of temperature excursion through a finite uniform thickness L submitted to a Dirac delta pulse was proposed as follows [24,26,32]:

(6) $$T_{fin}\left(z,\;t\right){|}_{z=0}=T_{0}+\frac{J_{0}}{e\sqrt{\pi t}}\left[1+2\overset{\infty }{\underset{n=1}{\sum }}\Gamma^{n}\;exp\left(-n^{2}\frac{L^{2}}{\alpha t}\right)\right]$$

**Figure 2:**
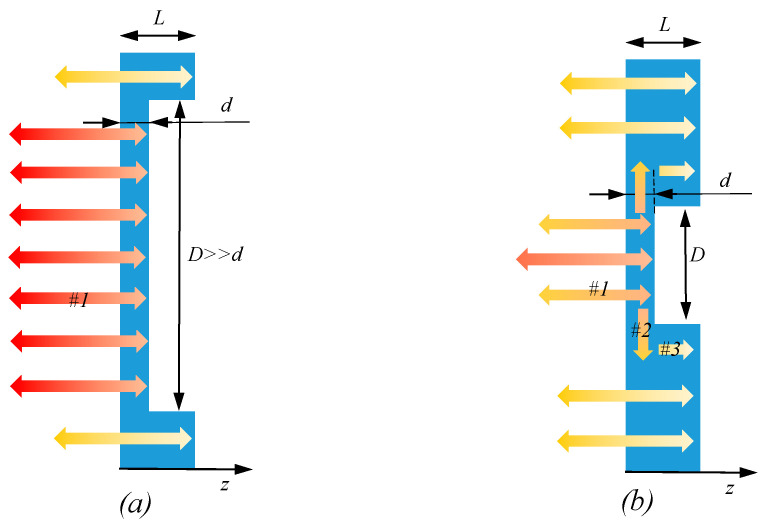
Comparison of the effect of defect (**a**) infinite and (**b**) finite lateral extension on heat diffusion, retrieved from [1].

The summation appearing in the bracket accounts for the effective multiple internal reflections, or reverberations, of the energy pulse between the air gap defect and the sample surface [26]. It is important to stress, the crucial assumption here is the infinite extend, D, of the defect along the lateral direction compared to its depth, d or D≫d [33] (see Figure 2a). In general, through IR thermographic NDT for corrosion detection, it has been mainly tried to apply one-dimensional (1D) models assuming that transient thermal processes occur independently in sound (non-defective) and defect areas [34]. It has been mentioned in the literature that the lateral extension of disk-shaped defects in steel material, D, should be about six times larger than the sample thickness, L, for the effective assumption of 1D heat transition [35]. Moreover, as a general agreement between practitioners of thermal NDT for sub-surface defect detection, the defect detectability of active thermography is generally considered reliable when the defect aspect ratio Dd exceeds two [33].

The lateral extension of a real corrosion defect, however, can be finite. As result, the incident heat front traveling above the defect will deviate laterally (see Figure 2b).

As shown in Figure 2b, this lateral heat diffusion results in three-dimensional heat transfer through medium, enhancing the diffusion over the defect (arrow 2) [26,36,37,38]. In fact, compared to Equation (6), the lateral diffusion of heat incident towards defect edges will result in less energy incident accumulation at the centre of the defect and, consequently, less temperature record at the surface over the defect. An analytical model of heat diffusion over defect, $T_{def}$, was proposed in order to take into account the reduction in thermal incident due to lateral thermal diffusion associated with defect finite lateral size, D [33]; see Equation (7). It is assumed that the defect edge acts as a heat sink transferring the heat incident from localised high-temperature area above the centre of the defect towards low-temperature area of defect underneath in order to establish the steady-state thermal equilibrium (arrow 3). It is then assumed that the associated reduction in heat over the defect is proportional to defect diffusion distant D2 as follows:

(7) Tdef(z, t) |z=0=T0+J0eπt[1+2∑n=1∞Γn exp(−n2d2αt)](1−exp(−(D2)24αt))

Defect detection by means of active pulse thermography (PT) experiments consists of a specimen subject to a relatively short energy pulse, and then recording the temperature evolution curves in transient mode (temporal). However, in practice, only producing quasi-Dirac pulse is possible. As a result, the square pulse of width t and amplitude A accounts for the most practiced waveform in PT applications [27,28]. However, the pulse width can be varied from an approximate ~2 ms (quasi-Dirac pulse) to a couple of seconds (long square pulse) [1].

The 1D classical heat conduction solutions, which describe square-pulse heating, $T_{sq}$, of a plate with adiabatic boundary conditions, are as follows [30,39]:

During energy stimulation:

(8) $$T_{sq}\left(z,\;t\right)\;{|}_{z=0}=T_{0}+\frac{QL}{k}\left[F_{O}+\frac{1}{3}-\frac{2}{\pi^{2}}\overset{\infty }{\underset{n=1}{\sum }}\frac{1}{n^{2}}exp\left(-n^{2}\pi^{2}F_{O}\right)\right]$$

After energy stimulation:

(9) $$T_{sq}\left(z,\;t\right)\;{|}_{z=0}=T_{0}+\frac{QL}{k}\left[F_{Oh}+\frac{2}{\pi^{2}}\overset{\infty }{\underset{n=1}{\sum }}\frac{1}{n^{2}}exp\left(-n^{2}\pi^{2}F_{O}\right)\left(exp\left(n^{2}\pi^{2}F_{Oh}\right)-1\right)\right]$$

where FO=αtL2 and FOh=αthL2 are the dimensionless Fourier numbers, respectively, for, after and during stimulation. $t_{h}$ is the energy pulse duration. Q is the absorbed energy density $\left(W/m^{2}\right)$.

Calculation of temperature difference for spatial domain over temporal domain is considered the most exercised IR NDT method in order to enhance subsurface defect visibility. This enables quantitative extractions of information, such as defect depth, size and thermal properties [26,31,40]. Careful consideration of a non-defective as the reference, however, remains challenging as, in a real-life case of corrosion (metal loss), identifying a non-defective or reference may not always be possible. Through some recent works [22,23], authors tried to address this issue by introducing a method known as Dynamic Reference Reconstruction (DRR). The motivation behind this method was the relative coexistence of reference (non-defective) and defective areas in proximity of each other, meaning a non-defective area can be considered as defective based on its contrast intensity level or, in contrast, a defective area can be hidden in a non-defective surrounding due to high similarity of their intensities. Accurate identification of defective and non-defective areas of test surface is an essential prerequisite of defect depth and size characterisation. In the current work, the aim has been to predict defects’ dimensions. Historically, this has been conducted by using key features of thermal contrast evolution time-series over defects, e.g., the maximum contrast value and the time the contrast trend reaches its maximum. Here, first, it was crucial to find an approximation model for cold-pulse active thermography. Then, the model was used to compare the experimental contrasts and find the closest similarity to their equivalent model.

## 2. Materials and Methods

**Figure 3:**
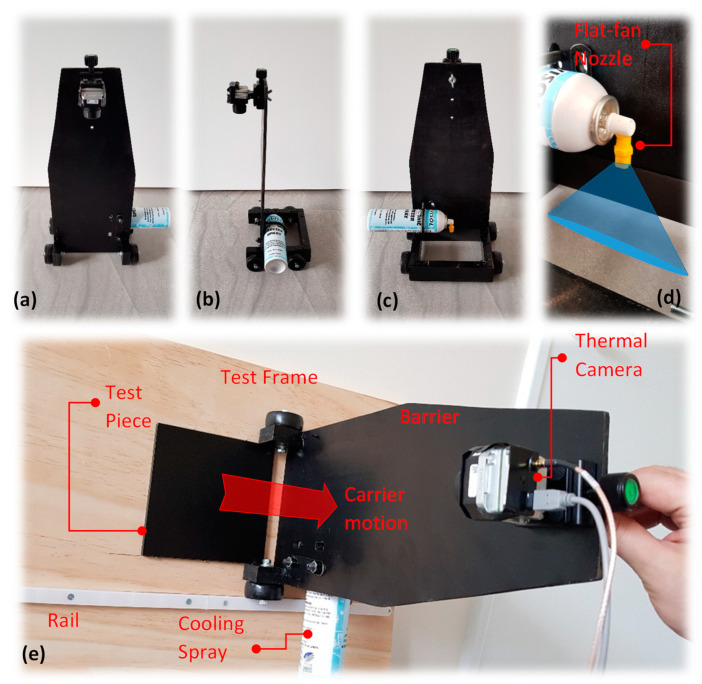
(**a**) Carrier front view; (**b**) carrier side view; (**c**) carrier back view; (**d**) cooling medium spray with customised flat-fan nozzle; (**e**) experimental setup showing carrier motion over the test piece.

As the use of cold stimulation instead of common heat sources is of interest here, a commercial cooling medium known as freezing spray consisting of R134A-based propellant was considered as the stimulation source. Unlike optical energy sources in which the thermal camera field of view is unobstructed, the cooling method can be disadvantageous due to the surface blocking effect of the cold stimulation while in operation. As result, a prototype was designed and introduced through previous works [22,23]. In this experimental setting, both the thermal camera and the cooling spray reservoir (can) are accommodated alongside a carrier and separated by a barrier, which holds the camera and prevents escaping cold burst (noise) to disrupt the camera view; see Figure 3. The components’ arrangement has been configured in a way that spraying action can be manually performed above the test surface while the carrier passes over the test piece surface, immediately exposing the stimulated surface to the camera; see Figure 3e. The camera transfers the video signals to a recording software. A test frame accommodates the test piece in a fixed position, while incorporating a guide rail into the test frame ensures linear motion across the test specimen; see Figure 3e. In order to prevent nonuniform cooling of the surface at its best, a modified flat-fan jet-spray nozzle is used. Flat-fan jet nozzle sprays the cold burst from a very narrow slot, creating a quasi-linear cross-section at the surface of test piece; see Figure 3d.

The thermal camera used for the purpose of this work is an uncooled long-wave infrared (LWIR) FLIR TAU2 640 thermal camera characterised with 640 × 512 pixel output resolution, 17 μm pixel size and the 7.5−13.5 μm spectral operating band. This device is equipped with a 19 mm wide field of view (WFOV) lens with $32^{{}^{\circ}}\times 28^{{}^{\circ}}\;\left(h\times v\right)$. A 19 mm lens is found to be optimal in terms of providing relatively wide coverage of the surface with the minimum image distortion. The recording output specification for the mentioned device includes recording video signals at 30 Hz nominal rate (29.97 Hz NTSC and 25 Hz PAL) in 8-bit (analogue) colour level. Experiments are performed under ambient temperature and pressure.

**Figure 4:**
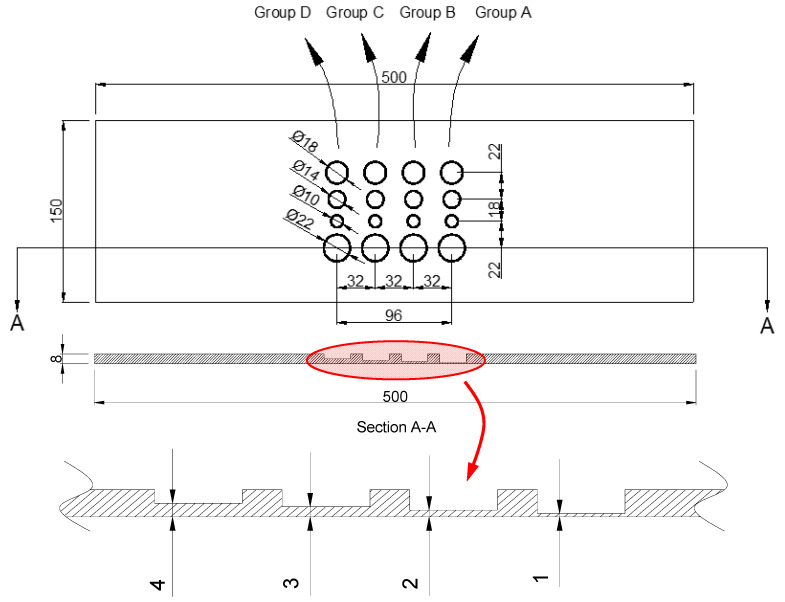
The test piece and the defect arrangements and dimensions.

The test piece considered for this research is manufactured from AS/NZS 1594—HA 250 structural mild carbon steel (see material properties in Table 1) of 500(L)×150(W)×8(t) mm dimensions. Four groups of sizes and depths of flat-bottom circular blind holes are drilled across one side (hidden side) in order to replicate the metal loss defects; see Figure 4 and Table 2. As shown in Figure 4, the test piece surface is covered by low-sheen black paint. This was carried out to reduce the possible external reflection from the surroundings. However, we found that the cold stimulation using cooling medium is less prone to creating reflections on a bare metal surface in comparison with heat pulse sources, e.g., flash lamps. Moreover, the reason for the extended length of test sample is to allow sufficient distance for the carrier and the cooling stimulation to be initiated before passing over the defects, as well as sufficient distance after the defect to fully pass over the last row of defect. This will ideally create a full field of view to capture the complete image sequence from the entire defective area.

In this work, each experiment consists of manually releasing (spraying) cooling medium during the transition of carrier from a non-defective area way behind the defective region to a sufficiently distant area after defective region. This motion must allow the camera to fully capture the first row of defects entering the field of view and the last row of defects exiting the field of view. As result, the assurance of constant linear travelling speed in order to guarantee the most uniform stimulation possible, as well as equal exposure of test piece surface to the thermal camera, is paramount.

**Figure 5:**
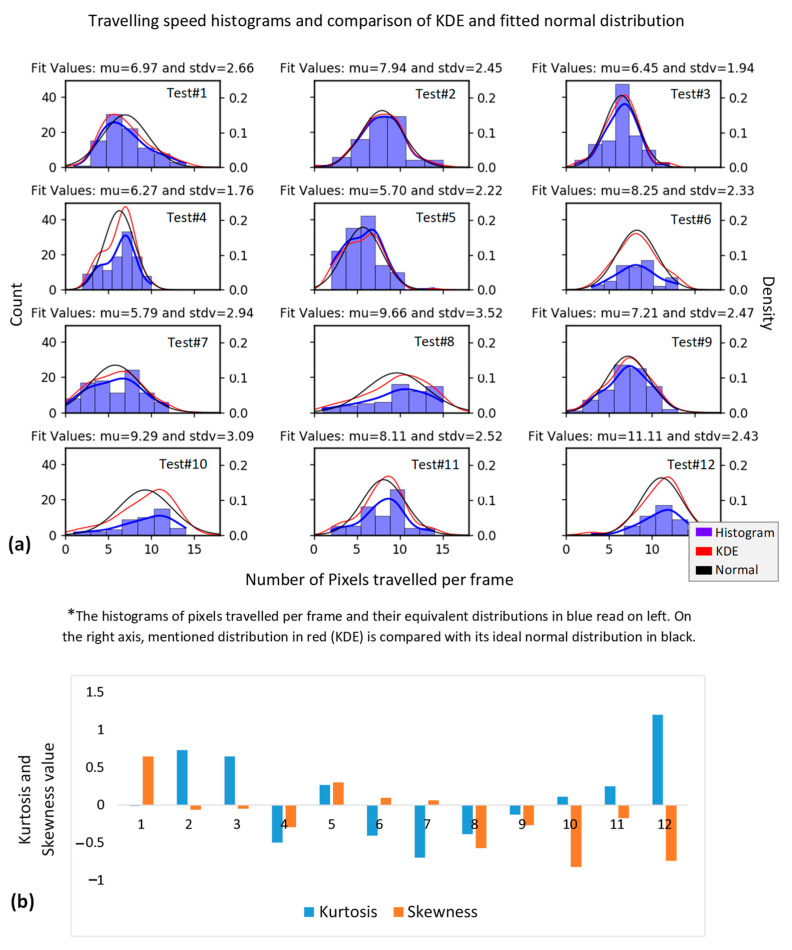
Statistical parameters representing the uniformity and consistency of the carrier speed. (**a**) Shows the histogram of test speeds and their equivalent distributions for 12 tests and (**b**) represents Kurtosis and Skewness values calculated from each distribution.

Multiple experiments were conducted, at which a full run of carrier passing over the test piece, exposing defective region to camera, occurred. Histogram and Kernel Distribution Estimation function (KDE) of carrier speed in unit of pixel per frame for each test was estimated to select the most acceptable test conditions and results. The measure for the most acceptable test conditions was considered to be the least skewed and the highest positive kurtosis of test speed distributions. Skewness is the measure of dataset symmetry or lack of symmetry, with skewness = 0 defining the normal (symmetrical) distribution. Kurtosis is the measure of dataset sharpness, with positive kurtosis representing the sharp (unique) distribution of a dataset, negative kurtosis representing the flat (uniform) distribution of a dataset and the zero kurtosis corresponding to the normal distribution (based on Fisher definition). Moreover, for each test, the speed distribution mean μ and standard deviation stdv were calculated. The approximately similar test speed mean and lower standard deviation were added to the selection criteria. Figure 5 shows an example of a set of 12 experiments and their statistical parameters of travelling speed. In Figure 5a, the histogram of pixels travelled per frame for each test has been presented. On the right axis, the equivalent KDE is compared with the idea of normal distribution of each experiment in order to highlight undesired distribution characteristics, such as skewness or bi-modality. Moreover, Figure 5b represents the estimated kurtosis and skewness for ach test. In this case, tests number 2, 3, 6, 9 and 11 showed more consistency.

## 3. Experimental Results and Discussion

### 3.1. Image Data Preprocessing

Information acquired using infrared cameras are generally converted into visible images by assigning colour intensities to each infrared energy level corresponding to either electromagnetic flux or the exact temperature (through radiometry). The result is called a thermogram, in which each pixel represents the evolution of temperature or electromagnetic flux in time [42]. In the current experimental setup, additional to the time transition, each image includes pixels for which spatial (location) features vary in each frame due to the camera motion. Therefore, each frame is required to be sorted in a raster-like array in which pixels preserve their spatial parameters along the temporal direction. This was achieved by stacking all frames in order to produce a 3D array of frames over exposure time. Image averaging of multiple tests has been strongly recommended through the literature in order to increase the signal-to-noise ratio [30]. Here, for various experimental configurations that will be addressed in a later section, the image averaging was conducted.

**Figure 6:**
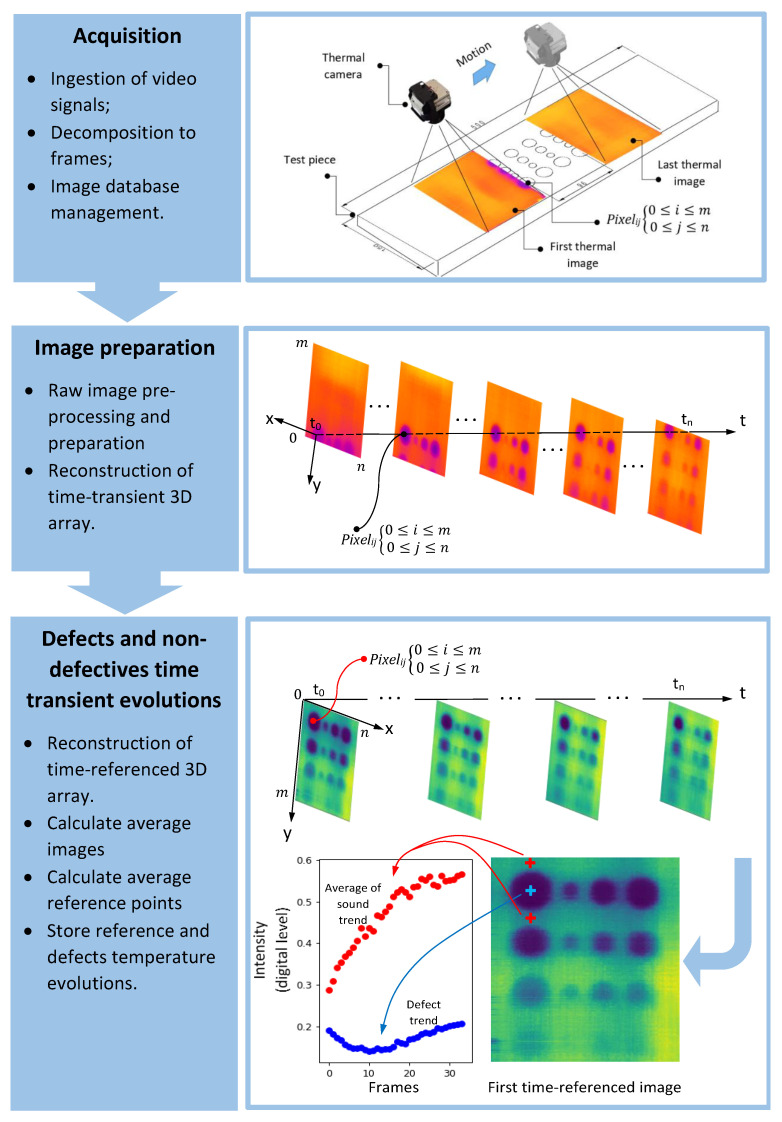
Experimental data ingestion, preprocessing and analytic pipeline steps.

Figure 6 represents a preprocessing pipeline which includes three steps: 1—the data acquired through thermal videos are preprocessed and decomposed to individual frames based on camera native frame rate; 2—a 3D array containing pixels’ spatial information over the time of experiment is reconstructed. Each pixel is characterised with a “*reveal time*” equivalent to when that specific pixel enters the camera field of view and an “*exit time*” when that pixel exits from the camera field of view. The difference between reveal time and exit time is known as “*test window*”. Test window is inversely proportional to test speed. Mentioned time characteristics for each pixel can be different due to the moving experimental setup exposing a different segment of test surface (group of pixels in each frame) at each point in time. As a result, in step 3, all pixels’ time transient evolutions are referenced from time perspective to $t_{0}=0$. The result is a sequence of images known as “*time-referenced images*”, in which each pixel co-ordinate is fixed. The indices i and j correspond to the co-ordinates of each point of interest (pixel) along time-referenced thermograms. For each defect, a point over the centre of defect (pixel marked with a blue +) and a set of sound (non-defective) points in the proximity of that defect are selected (pixels marked with a red +) in order to further calculate the contrast trends.

### 3.2. Temperature Evolution in Time

The analysis of IR thermographic corrosion detection has been mainly performed by applying a one-dimensional (1D) solution of Fourier equation for different excitation wave forms. Such analysis assumes that transient thermal evolutions occur independently in non-defective and defective areas. In order to characterise the corrosion defects using cold stimulation, first, an analytical model representing cooling wave form is required. The 1D analysis of corrosion detection by optical heating mainly involves using the classical heat conduction solutions, which describe either flash (Dirac delta pulse approximation) or square-pulse heating of a plate under adiabatic boundary conditions [30,34]. For the current experimental design, which is the case of moving stimulation source, the approximation of square pulse for the cooling stimulation was considered; see Equations (8) and (9). Then, the consistencies with the classical 1D square-pulse model, as well as multiple analytical and experimental simulations reported through the literature, has been investigated and, finally, the shortcomings of the 1D solution are addressed. The important assumptions here are:

(a) Heat diffusion in solid occurs on a pure conduction basis;
(b) The cold flux can be described as a square pulse characterised by the maximum density of absorbed power density Q and the stimulation duration $t_{stm}$;
(c) Adiabatic conditions can be accepted, meaning there is no energy exchange from both front and rear surface;
(d) Boundaries between the host material and air-filled defects can be regarded as adiabatic, meaning Γ≈1;
(e) First internal reflections, or reverberation, of the energy pulse between the air gap defect and the sample surface are considered to be the most dominant, meaning n=1 in Equations (8) and (9).

It is important to stress here, the output of the thermal device being deployed in this work is 8-bit analogue images in which temperature values for each pixel are translated to colour intensity between 0 and 256 or 0 and 1 (normalised greyscale image). As result, here, it is avoided comparing the experimental result with the 1D square-pulse model from a temperature value point of view, as the temperature curves are simply colour intensities adjusted based on surrounding intensities. Normalised or running contrast has been commonly used for the purpose of non-destructive testing for defect detection in thermography methods:

(10) $$\Delta T_{Run}=\frac{\left(T_{d}-T_{s}\right)}{T_{s}}$$

The index s is associated with a reference (sound or non-defective) area and index d is associated with a pixel located on the centre of a defect at the surface. Such normalisation is useful as, first, it removes the dependency to the absorbed energy value, which, in the case of cooling spray, is difficult to measure and, second, working with contrast curves compensates for intensity adjustment occurring in nonradiometric camera output images.

**Figure 7:**
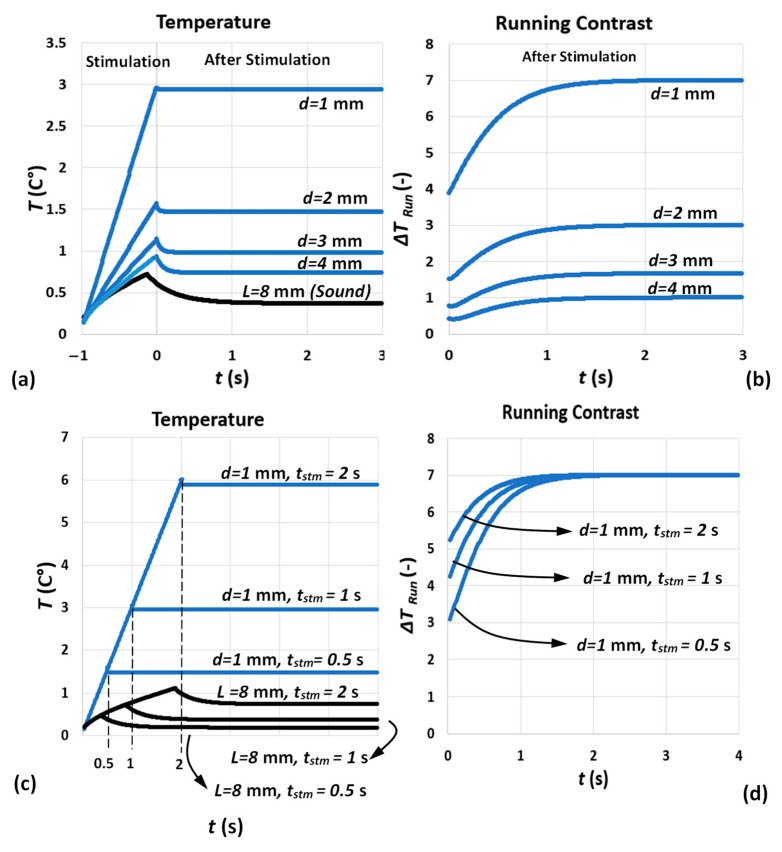
(**a**,**b**) 1D temperature and running temperature contrast defects with diameter D=22 mm and various depths, d, on an L=8 mm thickness mild steel specimen subjected to a square-pulse stimulation of duration $t_{stm}=1$ s. (**c**,**d**) 1D temperature and running temperature contrast of a defect with diameter D=22 mm and d=1 mm on an L=8 mm thickness mild steel sample subjected to square-pulse stimulation of duration $t_{stm}=0.5,\;1\;\mathrm{and}\;2$ s. The pulse absorbed power density is assumed $Q=10\;\mathrm{kW}/m^{2}$.

Figure 7 illustrates the 1D analytical solution of square-pulse stimulation (see Equations (8) and (9)) considering the above-mentioned assumptions. Figure 7a,b show the temperature and running contrast trends for various defect depths subjected to a square pulse of duration $t_{stm}=1\;s.$ Figure 7c,d show the same trends for various $t_{stm}$, while the depth is unchanged. As shown, only the after-cooling processes in running contrasts are considered due to the present experimental setup allowing only observation of the specimen surface immediately after cooling. In order to present a more familiar heating contrast curve to the thermography community and, eventually, using more familiar terms, such as contrast peak and decay, from here we multiply all experimental contrast curves by −1 and use $t_{stm}$ instead of $t_{h}$ in Equations (8) and (9), which is representative of stimulation duration regardless of its heating or cooling mechanism. Some important information from temperature and contrast evolution signal can be extracted, which is addressed elsewhere [30,34,43]:

(a) Based on a 1D square-pulse model, both maximum (end of stimulation) and minimum (a long time after stimulation) temperatures depend on pulse duration, absorbed energy of the pulse and defect depth; see Equation (11). However, the ratio between maximum and minimum is strictly proportional to pulse duration and defect depth; see Equation (12). In practice, the specimen temperature decreases slowly to the ambient level, meaning the contrast curve decays to zero. This is due to 3D heat diffusion.
(b) A shorter pulse tends to generate a greater ratio of maximum minimum temperature due to the fact that, in short pulse, only the near-surface layer of medium is being stimulated and the energy dissipates faster and stronger after a shorter pulse [30]. Table 3 shows some of the estimated temperature characteristics derived from 1D square-pulse equation. It is known from Equations (11) and (12) that the ratio between $T_{max}$ and $T_{min}$ in square pulse is mainly controlled by the pulse duration and it is independent of Q. However, for deeper defects, not only the decrease in $t_{stm}$, but also increasing depth has an exponential effect on significant increase in ratio, m; see the highlighted comparison in Table 3.
(c) Both maximum temperature and maximum temperature contrast (contrast peak) occurrence time tend to be earlier for longer pulse durations compared to shorter pulse [43].
  (11) $$\begin{array}{l} T_{min}=\left(\frac{QL}{K}\right)Fo_{stm}\\ T_{max}=\left(\frac{QL}{K}\right)\left\{Fo_{stm}+\frac{2}{\pi^{2}}\overset{\infty }{\underset{n=1}{\sum }}\frac{1}{n^{2}}\left[1-exp\left(-n^{2}\pi^{2}Fo_{stm}\right)\right]\right\}|n=1 \end{array}$$
  (12) m= TmaxTmin=1+2π2Fostm∑n=1∞1n2[1−exp(−n2π2Fostm)]|n=1

**Figure 8:**
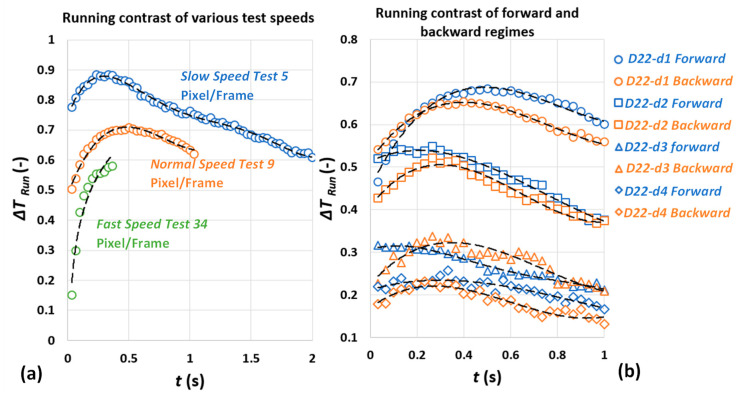
(**a**) Average running contrast of D=22 mm, d=1 mm defect for various test speeds; (**b**) average running contrast of D=22 mm defects with different depths under forward and backward regimes.

In order to compare the experimental results with both 1D and 3D square-pulse analytical and experimental results of the heating process and explore the consistencies and differences, various test regimes are performed. Figure 8 represents average running contrast of the experimental results under various test regimes. A polynomial fit of order 4 fitted to the data only for the purpose of visualisation. Figure 8a shows the average running contrast of three tests for defect D=22 mm , d=1 mm under three different test speed regimes. Three test speed regimes of “*Fast*”, “*Normal*” and “*Slow*”, respectively, equal to 34 pixel/frame, 9 pixel/frame and 5 pixel/frame were applied. As shown in Figure 8a, the contrast evolution observation window varies due to the test speed differences. The purpose of applying different test speeds is to provide various stimulation times and observe the effect of various stimulation times on key thermal contrast characteristics (i.e., contrast peak amplitude and contrast peak time).

Figure 8b also shows the comparison of average running contrast of six tests for each defect of D=22 mm , d=1 mm to d=4 mm under two different test direction regimes. Two test direction regimes include the “*forward*” tests where the shallowest defects (the closest to the surface, i.e., d=1 mm) are the first row being subjected to the cold pulse and the “*backward*” tests where the first row of the defect subjected to the cold pulse are the deepest (d=4 mm). This is performed by simply rotating the test piece for different sets of device operation. All tests in the mentioned test direction regimes are performed at approximately 9 pixel/frame speed. This was carried out in order to, first, assure the defect characterisation process independency of test direction and, second, study the cold-pulse behaviour in a moving energy source scenario. For the mentioned two test direction regimes, the available contrast observation window was estimated to be about 1 s.

Two important characteristics of various temperature contrasts are known to be contrast peak amplitude and contrast peak occurrence time.

#### 3.2.1. Running Contrast Peak Time

The temperature and temperature contrast evolutions generally include, first, increase in temperature over the defects due to reflection of energy, which is disrupted by defect, and then dissipation or decay (increase in case of cooling) of temperature back to ambient level due to 3D lateral heat diffusion. As shown in Figure 8a, contrast trends for a point over defect D=22 mm , d=1 mm reaches its maximum slightly earlier for longer pulse (slower test speed). For the fast speed setup in which the pulse is very short, no information from contrast peak amplitude and its occurrence time is available due to limitation of the surface observation window. It is, however, expected that short pulse reaches its maximum at a later time compared to normal and slow configurations, according to Section 3.2.

In Figure 8b, contrast trends for similar defects sizes in either forward (d=1 mm defects are first to receive cooling pulse) and backward (d=4 mm defects are first to be exposed to the cold pulse) test direction regimes are not significantly different. The contrast peak occurrence time is known to be proportional to pulse duration [30,35,43]. It is important to mention, the current experimental setup does not guarantee that the time that a pixel over a defect first appears to the camera is exactly equal to the end of cooling time in the 1D square-pulse model. In fact, it can be said that the reveal time in which the first pixel and its associated temperature/colour intensity is recorded by thermal camera or $t_{reveal}$ is always greater than analytical stimulation time or $t_{reveal}>t_{stm}$.

#### 3.2.2. Running Contrast Peak Amplitude

In the 1D square-pulse model, the contrast peak amplitude is inversely proportional to defect depth, while it is directly correlated to absorbed energy density Q. In case of running contrast, however, the dependency to Q is no longer relevant. It has been mentioned elsewhere that the contrast peak amplitude is independent of pulse duration $t_{stm}$ [43]. In Figure 8a, the running contrast peak amplitudes happen to vary for various cooling durations. It is expected that a longer duration of pulse generates higher intensities in the contrast image, despite the fact that running contrast amplitude of constant depth in the 1D model (see Figure 7d) is not dependent on cooling duration. In order to reject the proportionality of experimental contrast peak amplitude to cooling duration, the ratio of contrast peak amplitudes under various test regimes (speed and direction) can be compared. It is expected that the contrast peak ratio under different cooling durations for similar defect depths remain unchanged and equal to one. For that reason, the comparison of contrast peak amplitude ratios for slow against normal (test speed regimes) and forward against backward (test direction regimes) for various defects was considered. Here, it is assumed that, for defects with no visible contrast peak, the earliest value of contrast curve is sufficiently close to its equivalent contrast peak value in the 1D model.

**Figure 9:**
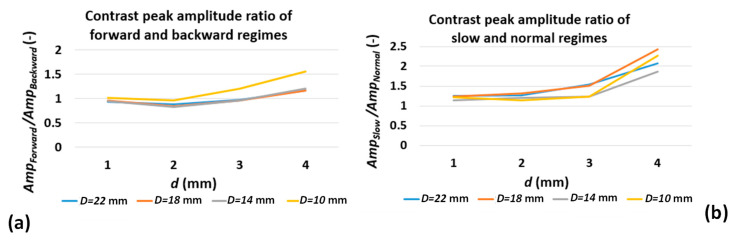
Contrast peak amplitude ratio of (**a**) various test direction and (**b**) various test speed regimes for various defect diameters and depths.

Figure 9 shows the ratio of defect contrast peak amplitude for two different testing regimes. Figure 9a shows the contrast peak ratio comparison from the test direction point of view and Figure 9b shows the same ratios for different test speeds (equivalent to different stimulation time). In Figure 9a, the contrast peak ratio of group of similar diameter defects and various depths are almost constant and close to unity yet deviate from constant ratio when the defect aspect ratio Dd, decreases. This means that the test direction does not affect the contrast peak amplitude (at least for the larger defect aspect ratio). In Figure 9b, the ratios of running contrast peaks for similar diameter defects and various depths are also almost constant but not equal to unity. Similarly, the ratio trends deviate from constant for deep defects. These findings are important as they reinforce that, across both experiment regimes, the first-order dependency of contrast peak on defect depth (at least in case of shallow defects) is preserved.

However, the contrast peak amplitude independency from stimulation duration derived from the 1D model has not been satisfied in Figure 9b because the contrast peak ratios for the group of same diameter defects are not equal to unity. Knowing all of the above-mentioned conclusions is critical as, first, it demonstrates that contrast curves generated from analogue image data with temperature values translated to colour intensities are still reliable means for characterising defects. The key characteristics of contrast curve, e.g., relative amplitudes of peak contrasts for various defects and contrast peak occurrence time are not affected by contrast enhancement. More importantly, it shows that the complex 3D heat diffusion known as decay process as a result of defect-limited lateral extension can contribute to change in contrast peak amplitude, as well as various stimulation durations. The detailed discussion of such phenomena is out of the scope of the current work.

### 3.3. Proposed Analytical Model

Some important characteristics of defect temperature contrast derived from experimental results demonstrate to be in good agreement with the 1D square-pulse model. These include:

1. First-order effect of pulse duration on temporal characteristics (contrast peak occurrence time) of both temperature and contrast peak;
2. The first-order effect of depth on amplitude of contrast peak.

However, the 1D square-pulse model is unable to describe the 3D (lateral) energy diffusion as a result of defect finite lateral extension. This phenomenon can be observed in the form of contrast decay following the contrast peak and the 1D model does not take this characteristic into account.

Here, a simple analytical model is proposed. This analytical approximation model complements 1D square pulse with an additional term representing the lateral energy dissipation associated with the defects’ lateral extension (diameter) as an approximation for cooling stimulation response. Such a model can then be used to predict the defect depth and diameter by comparing it with experimental results. A simplified analytical term representing 3D lateral heat diffusion was previously introduced for flash-pulse thermography model [33]; see Equation (7). In this work, the mentioned term was multiplied to the 1D square-pulse running contrast with the assumption that the evolution of the thermal contrast over the centre of a defect is limited by the rate of lateral diffusion of heat from the centre to the defect edge; see Equation (13). For a circular defect of diameter D, the diffusion distance is D2.

(13) ΔTRun3D=(Td−Ts)Ts·(1−exp(−(D2)24αt))

**Figure 10:**
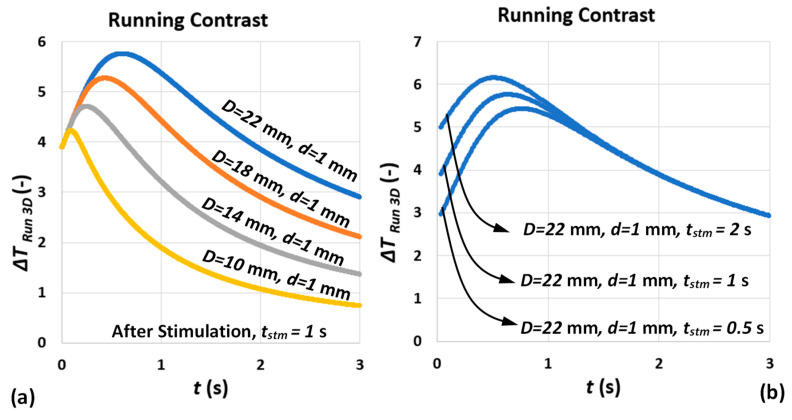
(**a**) Analytical (3D) running contrast defects for various diameter, D, on an L=8 mm thickness mild steel specimen subjected to a square-pulse stimulation of duration $t_{stm}=1$ s. (**b**) Analytical (3D) running contrast of a defect with diameter D=22 mm and d=1 mm on an L=8 mm thickness mild steel sample subjected to square-pulse stimulation of duration $t_{stm}=0.5,\;1\;\mathrm{and}\;2$ s. The pulse-absorbed power density is assumed $Q=10\;\mathrm{kW}/m^{2}$.

Figure 10a shows the analytical model of running contrast for different defect diameters and depth of d=1 mm. The effect of defect lateral extension on both contrast peak occurrence time and amplitude is consistent with abundance of analytical and experimental results of pulsed thermography reported through the literature [26,33]. Figure 10b represents the effect of pulse duration on contrast evolution after stimulation. The dependency of contrast peak occurrence time on pulse duration mentioned earlier (see Section 3.2) has been satisfied with the longer the pulse, the earlier the contrast peak occurs. However, the contrast peak amplitude is no longer independent from pulse duration (according to 1D model) similar to what has been observed in experimental results; see Figure 8a.

**Figure 11:**
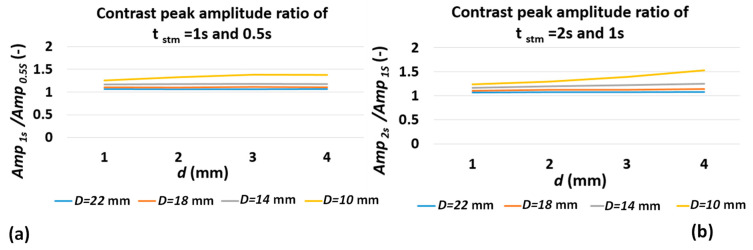
Contrast peak amplitude ratio of (**a**) 0.5 and 1 s and (**b**) 1 and 2 s pulse duration for various defect diameters and depths.

Figure 11 compares the ratio of contrast peak amplitude for various defect depths and diameters subjected to various pulse durations extracted from the proposed model. Comparing the results of Figure 11 and Figure 9b, one can observe a good agreement between analytical model and experimental results. The direct and first-order correlation of contrast peak amplitude to defect depth is preserved, while the effect of pulse duration on ratio of peak amplitude is insignificant (for larger defect aspect ratios). This means that, while, according to Figure 8a and Figure 10b, contrast peak amplitude is proportional to pulse duration for individual depths, which is unaddressed by the 1D square-pulse model, the ratio of contrast peak amplitude of consecutive defect depths for different pulse durations remains unchanged. Further, it can be concluded that the most significant contributor to contrast peak amplitude is still defect depth.

**Figure 12:**
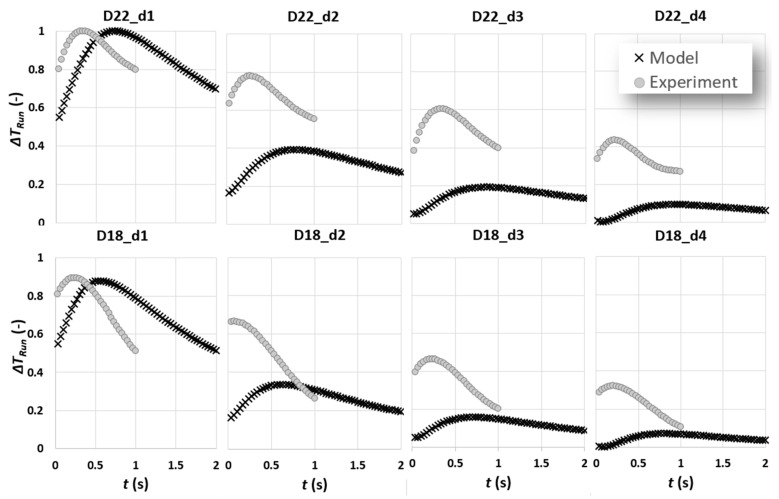
A comparison between experimental results and analytical model for defects of diameter 22 and 18 mm for various depths.

In Figure 12, the experimental and model results are compared. The experimental results are extracted from average contrast trends of six tests with approximately 9 pixel/frame test speed. A polynomial degree of 4 fit represents each experimental result trend. The time it takes the cooling burst to pass over one row of defects to another is estimated to be around 0.5 s. The same pulse duration is used to estimate the model results. For the purpose of consistency, both experimental and model results are scaled between 0 and 1. Important observations are as follows:

1. Except for the results of defects of depth 1 mm, there is an offset between experimental and analytic contrast peak amplitudes. It was found that this is due to the contrast adjustment process known as “*Automatic Gain Correction*” or “*AGC*” occurring in the camera software. In linear AGC, 14-bit digital data are transformed based on a linear transformation function to 8-bit colour intensities. The weakness of linear AGC is, however, quite pronounced in scenes characterised with bi-modal histogram of intensities in which some areas with very high or low intensities can be, respectively, over-enhanced or under-enhanced (which is exactly the case for subsurface defect detection) [22]. This can result in loss of important information, which, in case of a dynamic scene similar to what has been configured in this work, can translate to loss of key information from contrast evolution data. A detailed discussion of linear and nonlinear contrast enhancement in subsurface defect detection using cold thermal imaging has been addressed through authors’ previous works [22,23]. Here, the automatic mode of AGC using nonlinear transformation function is used, in which the entire intensity range available in 14-bit thermal data has been transformed to 8-bit colour intensity. The adjustment of contrast in each frame is heavily based on the available range of intensities. In thermal images of subsurface defects, the range of intensities is highly dependent on the presence of very dark (defect) and very light (reference) intensities. As a result, we compared the ratio of contrast peak for the model and experiments.

**Figure 13:**
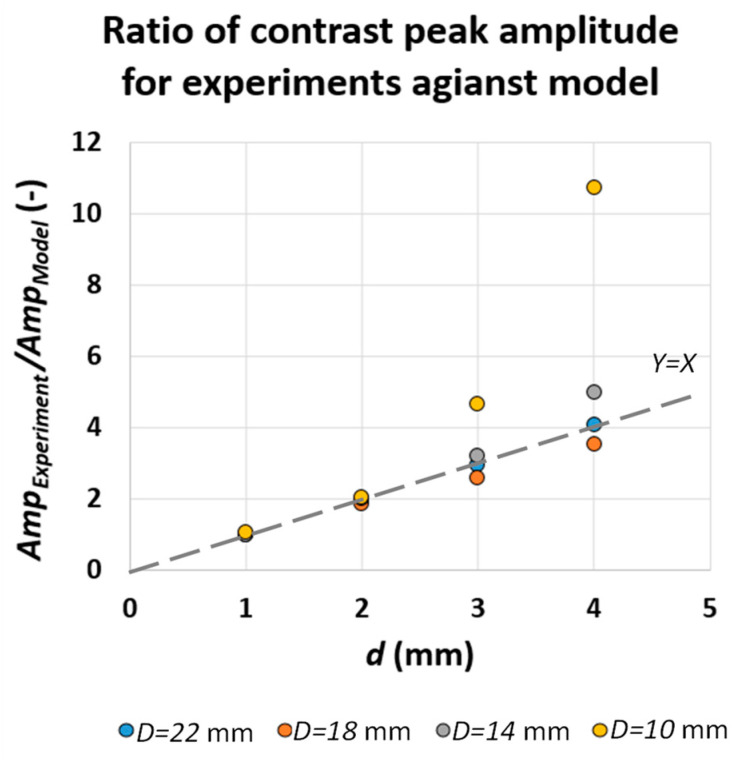
The ratio of contrast peak amplitude for experiments against model.

**Figure 14:**
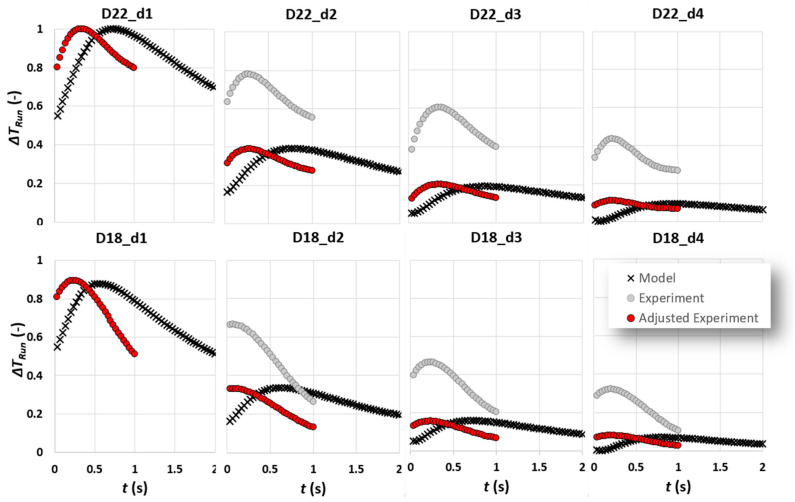
A comparison between experimental results, analytical model and adjusted model for defects of diameter 22 and 18 mm for various depths.

Figure 13 shows that there is a quasi-linear proportionality through an identity function of contrast peak ratio to defect depth, which is almost consistent for various defect diameters. The proportionality of contrast peak ratio to depth, however, deviates from linear identity trend when the defect aspect ratio significantly decreases. Using the result of this comparison, the experimental results are adjusted (scaled) to take into account the contrast enhancement performed in the camera software; see Figure 14.

2. For all experimental contrast evolutions, there is an offset in terms of contrast peak occurrence time in a way that the contrast peak occurs earlier than its equivalent model peak. This can be attributed to two phenomena: first, the fact mentioned previously addressing the latency of capturing experimental results as a result of current experimental setup or $t_{reveal}>t_{stm}$; second, the duration of complex cooling processes over a defect, which is simplified to a square pulse, might not be accurately measurable.

As shown in Figure 14, the first-order effect of defect depth on contrast peak amplitude is fully visible. In the meantime, the first-order effect of cooling pulse duration on contrast peak occurrence time is no longer significant compared to the effect of defect lateral extension (diameter) on the mentioned temporal characteristic. It is visible that, by reducing the defect diameter, the contrast peak occurs earlier, which is consistent with the multiple experimental and analytical findings in the literature.

Considering the above observations, the problem of defect depth characterisation can be reduced to measure the similarity between two offset time-series. One time-series represents the partially registered experimental contrast curve and the other one accounts for the complete analytical adjusted contrast curve belonging to the after-cooling stimulation period. In this work, a method of measuring time-series similarity known as “*Dynamic Time Wrapping*” or “*DTW*” is exercised.

### 3.4. Dynamic Time Wrapping for Defect Depth Prediction

**Figure 15:**
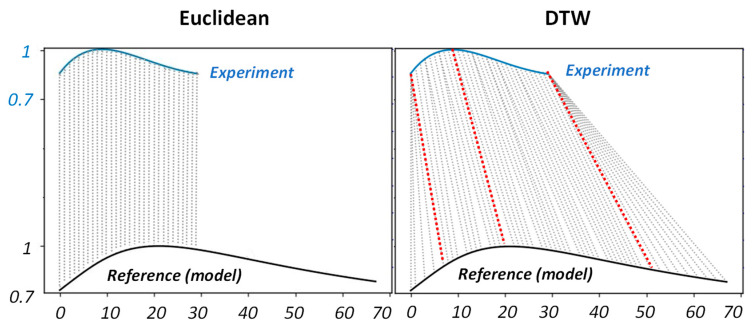
A comparison of Euclidean and DTW alignments of experimental and analytical model of running contrast curve for defect D=22 mm, d=1 mm.

There are a variety of methods dealing with time-series similarity investigations. Some methods, such as Euclidean distance metric or the mean absolute percentage error (MAPE), are based on point-wise calculation of the difference between two time-series. This enables such methods to be quite fast in computation; however, their applicability can be limited when two time-series under investigation are characterised with temporal or spatial offset or of different scales [44,45,46]. Dynamic time warping (DTW) is a well-known point-wise technique, which finds the temporal alignment that minimises Euclidean distance between aligned series regardless of their offset or scale. Figure 15 illustrates the fundamental differences between Euclidean and DTW in finding minimum distance between two experimental and reference (adjusted model) running contrast time-series for defect D=22 mm , d=1 mm.

As shown in Figure 15, DTW shows to be significantly more intuitive in aligning key points (connected via red dash lines) across two time-series. It is important to note here, we refused to interpolate the experimental time-series in order to achieve the same length as reference time-series. This is due to the fact that the experimental time-series has been considered a segment of reference time-series. It has been mentioned elsewhere [47] that scaling time-series to be in equal length does not add any benefit in the accuracy of DTW, while it is essential in Euclidean.

**Figure 16:**
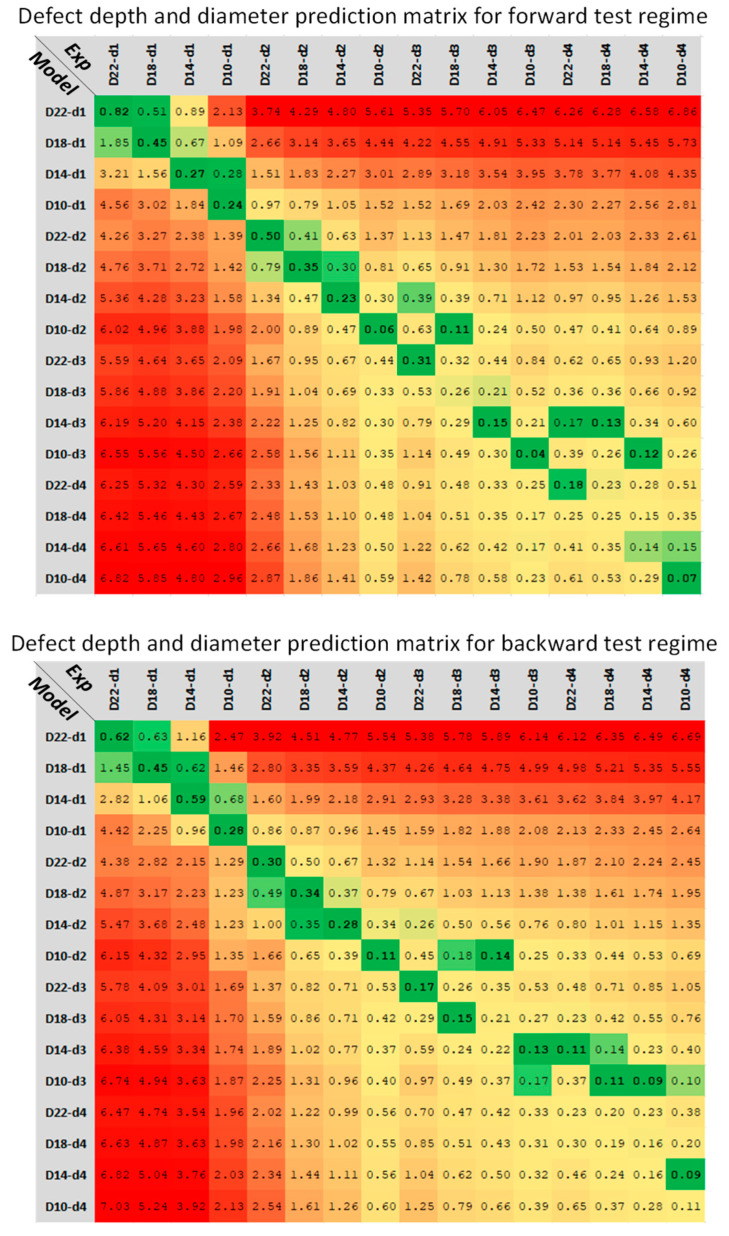
Defect depth and diameter prediction using DTW method for the forward and backward test regimes. Green color reflects the 10% percentile minimum range of similarity scores and change of colours towards red is equivalent to reduction of similarities (increasing similarity score) between experimental contrast and analytical model contrast time-series.

Figure 16 represents the predicted depth and diameter values for each defect by pairwise comparison of the experimental running contrast time-series with analytical running contrast model. Each cell in the prediction matrix contains the similarity score acquired from DTW calculation. Green cells highlight the 10% percentile minimum range of similarity scores. Ideally, the values located at the matrix diagonal should be the minimum for each defect (on the matrix header), meaning there is maximum similarity between defect contrast evolution and its equivalent model (on the matrix index). However, as is shown in Figure 16, by decreasing the defect aspect ratio, Dd, the prediction accuracy will decrease. This can be as the result of increased signal-to-noise ratio, in which the detection of defect becomes difficult and, consequently, an accurate characterisation unsuccessful. Moreover, the simple linear relationship presented in Figure 13, accounting for contrast enhancement performed by camera, is not valid when the defect aspect ratio decreases. Performing these experiments with a radiometric thermal device and extracting the running contrast curve from temperature data and not from contrast enhanced images may provide one with more accurate results.

Table 4 summarises some of the seminal works in the literature performed in order to detect and charactrise the metal loss defects in a metallic specimen. Note that the detection and characterisation limits are mentioned here regardless of their accuracies. Comparing the results of defect depth prediction acquired in this work with both classical heating flash-pulse and square-pulse experiments reported in the literature, we found that cold thermography can provide almost the same level of depth prediction as heating square-pulse thermography. The analysis of both flash and square-pulse heating procedures has historically shown that flash heating can be a more viable option to provide maximum temperature contrasts over defects but may deliver not enough energy to produce noticeable differential temperature signals, especially in thicker specimens.

Flash heating has shown a detecting capability of up to 10% material loss in steel samples with the test piece thickness up to 3 mm, while, in thicker samples (up to 5 mm thickness), the detection limit decline is up to 25%. In thicker metals, the phenomenon of lateral heat diffusion is responsible for dissipation of thermal evolution at the surface. As a result, it has been recommended that square-pulse heating can be a more suitable option in order to inspect thicker materials beyond 5 mm thickness, as it can deliver a higher rate of energy at a longer period of time. Based on multiple works found in the body of literature, while the defect detection up to 20% metal loss (for defect sizes larger than 20 mm) in thick specimens is overly achievable, a successful depth prediction of defect depth deeper than 50% of specimen thickness has been barely reported.

## 4. Conclusions

In this work, the previously introduced experimental setup incorporating cooling stimulation for the purpose of active thermography defect detection has been expanded in order to approximate the defect depth. An analytical model of heat diffusion was proposed based on the assumptions of cooling stimulation considered as square-pulse wave form and three-dimensional heat diffusion in solids proportional to defect lateral extension. Running contrast time-transient evolutions for each defect are extracted from sequences of thermal images. A time-series similarity measurement method known as dynamic time wrapping (DTW) is used to establish pairwise comparison between experimental and analytical contrast time-series [45,46]. The results of defect depth prediction have shown to be very promising and almost comparable with the result of heating square- and long-pulse defect detection and characterisation.

Where a pulsed thermography or, in general, active thermography using heating methods cannot be implemented, the cold thermography has shown that it can be a viable alternative. The final outcome of the proposed experimental setup has revealed that there is a good potential to predict the metal loss of up to 50%, even with using a less accurate nonradiometric thermal device and no advanced image processing method. The potential in situ application of cold active thermography in defect detection has been previously addressed. In this work, a more quantitative defect characterisation process has been explored and evaluated. It is, however, crucial to replicate such an experimental setup while incorporating a radiometric thermal device in which the thermal image pixel values can be translated to temperatures. The recent advances in the development of smaller size thermal devices with radiometric capabilities will enable one to replicate the cooling stimulation experimental setup without compromising the portability and in situ characteristics of the prototype.

## Figures and Tables

**Table 1:** HA 250 mild steel mechanical and thermal properties [41].

Chemical Composition		Mechanical and Thermal Properties	
Carbon, C	0.10–0.20%	Density	7.83 × 10^3^ (kg/m^3^)
Iron, Fe	98.81–99.26%	Tensile Strength, Yield	350 (MPa)
Manganese, Mn	0.45–1%	Thermal Conductivity	64 (W/m·K)
Phosphorous, P	≤0.040%	Specific Heat	434 (J/kg·K)

**Table 2:** The arrangement of depth and size of artificial defects.

Groups	A				B				C				D			
**D (mm)**	22	18	14	10	22	18	14	10	22	18	14	10	22	18	14	10
d * (mm)	1	1	1	1	2	2	2	2	3	3	3	3	4	4	4	4

D: diameter; d: depth; * the depth is the distance measured from the test piece surface.

**Table 3:** Some temperature curve key characteristics calculated from 1D square-pulse model for various defect depths and pulse durations. A deep defect (d=4 mm) subjected to a short pulse ($t_{stm}=0.1\;s$) demonstrates much larger min–max temperature ratio compare to other combinations of pulse duration and defect depth.

Groups	$t_{stm}=0.1\;s$				$t_{stm}=0.5\;s$				$t_{stm}=1\;s$				$t_{stm}=2\;s$			
d	1	2	3	4	1	2	3	4	1	2	3	4	1	2	3	4
$Fo_{stm}$	1.88	0.47	0.21	0.11	9.44	2.35	1.04	0.58	18.8	4.7	2.09	1.17	37.7	9.41	4.18	2.35
$T_{max}$	0.32	0.19	0.18	0.16	1.5	0.79	0.58	0.48	2.97	1.53	1.07	0.85	5.91	3.00	2.05	1.59
$T_{min}$	0.29	0.14	0.09	0.07	1.47	0.73	0.49	0.36	2.94	1.47	0.98	0.73	5.88	2.94	1.96	1.47
m	**1.10**	1.42	1.84	**2.22**	1.02	1.08	1.19	1.34	1.01	1.04	1.09	1.17	**1.00**	1.02	1.04	**1.08**

d: defect depth, FOstm=αtstmd2, α=k/ρC, k = 64 W/mK; ρ = 7830 $\mathrm{kg}/m^{3}$, C = 434 J/kg K, $Q=10\;\mathrm{kW}/m^{2}$.

**Table 4:** A summary of defect detection and characterisation limits in mild-steel specimens reported in the literature.

Year	Authors	Sample Thickness (mm)	Metal Loss Detection Limit (%)	Metal Loss Prediction Limit (%)	Method	Refrence
1996	Vavilov et al.	1.3	10%	25%	Flash pulse	[30]
1998	Grinzato et al.	4	20%	20%	Flash pulse	[48]
2010	Marinetti et al.	3	10%	10%	Flash pulse	[34]
2010	Marinetti et al.	10	20%	50%	Square pulse	[34]
2017	Almond et al.	6	20%	50%	Long pulse	[49]

## Data Availability

Not applicable.
